# A Jubilee Eviction

**Published:** 1897-07-24

**Authors:** 


### EDITOR'S LETTER-BOX.

[Our Correspondents are reminded that prolixity is a great bar to publication, and that brevity of style and conciseness of statement greatly facilitate early insertion.]

### A JUBILEE EVICTION.

WE publish the following curious communications from the House Visitor of the Royal Eye Hospital, Southwark, on the general principle of giving every man rope enough. It is clear, however, from what is therein contained that we were perfectly correct in what we said in the article which is assailed, and that the managers of this hospital did interrupt the work that they were appointed to perform, and did turn their hospital into a grand stand for the sake of letting Jubilee seats. The extent of the disturbance may be judged of from the fact that the official plan of the seats to be let stated that the windows were to be taken out, and showed between 900 and 1,000 numbered seats, besides standing places, while the total capacity of the hospital was such as to provide only 42 beds. We called attention to the matter, because we considered it a scandal that the interests of the patients should be so lightly considered; but we think that a still more important lesson is to be learned from the incident, which, in fact, well illustrates the evils of the special hospital system. Either the Royal Eye Hospital is wanted for the relief of ophthalmic cases which could not get the necessary relief elsewhere, or it is not wanted for that purpose. If it be a public institution which is necessary to the public well-being, then it is perfectly clear that the managers could not properly close it as they did for the purpose of making money on the occasion of the Jubilee; while if, as appears probable from the report of the House Visitor, the hospital is not so necessary, it seems to be a great pity that the institution is not permanently closed and the funds handed over to one of the large hospitals which must necessarily be always open, and where an adequate provision could be made at a much less cost. It is high time that the public recognised the evils and extravagances of certain special hospitals known as "one man" institutions. If at a time of Jubilee it is right to send "urgent ophthalmic cases" all the way to the Temperance Hospital in the Hampstead Road, what possible reason can there be for erecting such an institution as the Royal Eye Hospital in such close proximity as it is to Guy's and St. Thomas's Hospitals, and with the Westminster Ophthalmic Hospital just over the bridge? To those who know the true inwardness of these matters it is clear that the erection of many of the special hospitals in London has been determined by motives with which the interests of the patients have had but small concern.

MRS. J. E. COPE, secretary of the Royal Eye Hospital, Southwark, S.E., writes: By direction of the council of this hospital I offer you the opportunity and inquire if you intend to insert in your next issue the accompanying extract from the house visitor's report, which was read and adopted at this council's meeting on the 8th instant, and whether you will ensure it at least the same publicity as the malicious mis-statements which appeared in your paper of June 12th, 1897, under the heading "Jubilee Evictions."

ROYAL EYE HOSPITAL, SOUTHWARK, S.E.—SUMMARY EXTRACT FROM REPORT OF THE HOUSE VISITOR, READ AND ADOPTED BY THE COUNCIL, JULY 8TH, 1897.

In accordance with the recommendations authorised by the council the whole of the beds provided in this hospital (42, inclusive of the 14 hitherto closed) were at the service of the honorary medical staff during the whole of April and May. From the end of May admission of in-patients was curtailed, as far as possible without hardship to any, and during the third week in June the hospital contained but two in-patients. The eye of one of these two patients was in such a critical condition that it would have been injudicious to move him to any of the alternative beds kindly held available at King's College Hospital, the Royal Free Hospital, and the Temperance Hospital for urgent ophthalmic cases which might have been inconvenient to admit here during June. For Jubilee Day the two in-patients were accommodated in a room on the first floor of this hospital, and as neither was in such a condition that he could prudently use the window for seeing the procession, such window was freely placed at the disposal of the poor patient's wife and children. The work of the out-patient department was carried on without interruption throughout June, and its regular work was stopped on only one week day, June 22nd, which being a Bank Holiday was in accordance with the rules of the charity. For one week immediately prior to the Jubilee Day the south consulting room, and for two weeks the south private room were withdrawn from the out-patient service. This in no way impaired the thoroughness of the out-patient service, but caused some inconvenience to the officers conducting the same. The out-patient waiting hall was disarranged on Monday, June 21st, but on this day the attendance of out-patients was numerically so insignificant that no material inconvenience resulted. By half-past one p.m. on Wednesday, June 23rd, all the previous day's temporary fittings were cleared from the out-patients' waiting hall, from the south consulting room, and generally from the entire out-patient department, so that every usual facility was available for the smooth working of this department at the hour it opened on June 23rd, the day following the Jubilee Bank Holiday. Subsequently no time has been lost in reinstating the in-patient department ready for its usual work; but in view of completing the annual holidays of the nursing and domestic staff, towards which the month of June has been largely utilised, as well as for necessary hot water and electrical overhauling, it has seemed better to check rather than encourage the filling of the in-patient beds. The in and out patient departments of the hospital, the nursing and domestic staff, are now fully equipped, except so far as the fourteen beds provided in the Alexandra ward and the east second-floor ward are concerned. These are again closed until the Council feel warranted in opening them by the acquisition of increased support. The extra accommodation furnished in April and May, more than counterbalanced that curtailment of in-patients' accommodation which, in this Jubilee year, it was obviously advantageous to make in June, rather than in August and September.

